# The Cholecystokinin-B Receptor as a Candidate Diagnostic Biomarker and Translational Target for Precision Oncology in Pancreatic Cancer

**DOI:** 10.3390/cancers18213551

**Published:** 2026-11-05

**Authors:** Jill P. Smith

**Affiliations:** Department of Medicine, Georgetown University, Washington, DC 20007, USA; jps261@georgetown.edu

**Keywords:** tumor receptor, pancreatic cancer, nanoparticles, TCGA, biomarker

## Abstract

**Background/Objectives**: Identification of cell-surface receptors on cancer tissues that are absent in the corresponding normal tissue provide an opportunity for target-specific diagnostics and drug delivery. The cholecystokinin-B receptor (CCKBR) was initially overlooked as a high-priority biomarker for pancreatic cancer (PDAC) because comparisons were made between cancer and “normal adjacent tissue” from The Cancer Genome Atlas (TCGA). Since PDAC is highly invasive and significantly alters the tumor microenvironment, we now know the adjacent tissue is not normal in PDAC. **Methods**: A multi-cohort transcriptomic analysis was conducted to evaluate the true expression landscape of CCKBR in human pancreatic tissue. Bulk RNA-sequencing data were harmonized from the Genotype-Tissue Expression (GTEx) repository (*N* = 328) with true normal pancreas samples and TCGA Pancreatic Adenocarcinoma cohort (*N* = 178) primary tumors. Confirmation of CCKBR protein expression was performed using a human tissue array. The significance of a single-nucleotide polymorphism (SNP, rs1800843) that results in CCKBR mis-splicing was characterized. A biodegradable fluorescent polyplex nanoparticle (NP) was developed to selectively target the CCKBR and was tested using p48-Cre/LSL-Kras^G12D^ (KC) mice. **Results**: CCKBR transcripts exhibited a profound, binary-like upregulation in malignant tissue compared to normal tissue, achieving statistical significance (*p* = 10^−60^). The germline SNP of the CCKBR (rs1800843) was identified in ~40% of patients with confirmed PDAC. Immunoreactivity for the CCKBR protein was absent in normal human pancreas but increased in precancerous PanIN lesions and cancer. A fluorescent labeled CCKBR-targeted NP revealed uptake in the pancreas of KC mice and localized to the PanIN lesions. **Conclusions**: These data provide new insights into CCKBR, supporting its potential as a diagnostic biomarker and a therapeutic target in precision pancreatic oncology.

## Simple Summary

This work describes a highly specific marker for pancreatic cancer called CCKBR. Previously, this receptor biomarker was overlooked because scientists compared cancer tissue to “nearby tissue,” which they now realize is already altered by the disease. When compared to true healthy pancreas tissue, the CCKBR marker is almost exclusively found in cancer and precancerous cells. Because this marker is virtually absent in healthy pancreas tissue, it creates a potential target for treating the disease without harming normal cells. A biodegradable nanoparticle was developed that selectively binds to this receptor. This discovery clears up past confusion and establishes CCKBR as a unique tool for early detection and precise, targeted drug delivery in pancreatic cancer.

## 1. Introduction

Pancreatic ductal adenocarcinoma (PDAC) remains among the most lethal malignancies worldwide, with its global incidence and mortality rates consistently rising. Recent comprehensive epidemiological tracking demonstrates that the global burden of pancreatic cancer has expanded significantly, driven markedly by escalating metabolic risk factors, including high-fat diets, type-2 diabetes and a high Body Mass Index (BMI) [1,2]. This shifting metabolic landscape underscores the urgency of identifying robust diagnostic biomarkers and targeted risk-stratification models across diverse international patient populations.

The paradigm of modern oncology has fundamentally shifted from empirical, tissue-of-origin chemotherapy regimens toward precision medicine strategies guided by patient-specific molecular profiles [3]. Central to this clinical evolution is the discovery and validation of highly selective cell-surface receptor biomarkers. Because cell-surface receptors act as accessible physical portals bridging extracellular signals with critical intracellular cascades, they serve as unique, dual-purpose tools in precision oncology frameworks [4].

From a diagnostic perspective, cell-surface biomarkers provide an early, non-invasive window into tumor pathology. When engineered into targeted tracer compounds—such as radiolabeled small molecules or single-chain variable fragments—these receptors act as specific focal anchors for advanced molecular imaging platforms like Positron Emission Tomography (PET) or Single-Photon Emission Computed Tomography (SPECT) [5]. By employing specific radiolabeled probes [6], these techniques enable comprehensive, dynamic, and non-invasive mapping of biomarker expression and distribution within primary tumors and across metastatic sites. This functional visualization allows clinicians to evaluate biological processes at the cellular level earlier and with greater precision than conventional macro-imaging modalities, which rely strictly on changes in anatomical lesion size and can miss early-stage or microscopic tissue alterations.

Therapeutically, cell-surface receptors present a distinct molecular vulnerability that can be exploited to bypass the toxicities associated with conventional systemic treatments. High-affinity surface receptors can be selectively targeted using modern oncology toolkits, including monoclonal antibodies, small-molecule inhibitors, antibody-drug conjugates, and peptide receptor radionuclide therapies [7]. By pairing structural specificity with cytotoxic or radiotoxic payloads, these targeted medicines maximize tumor cell death while sparing non-target, rapidly dividing healthy cells. Clinical evidence confirms that patients receiving biomarker-guided, targeted approaches consistently show significantly improved therapeutic outcomes and reduced treatment toxicities compared to traditional empirical frameworks [3].

The primary prerequisite for a successful precision oncology target is a wide, dependable therapeutic window: the candidate receptor must demonstrate minimal baseline expression in vital organ systems coupled with robust, persistent overexpression across the targeted tumor population. Identifying and exploiting these binary transcriptional switches remains a core priority in developing effective therapies for historically treatment-resistant solid tumors.

The cholecystokinin B receptor (CCKBR) serves as a critical, dual-action biomarker and targeted therapy avenue in precision oncology, particularly for pancreatic ductal adenocarcinoma (PDAC). A major historical factor contributing to the research community’s delayed recognition of *CCKBR* as a high-priority biomarker is a pervasive structural artifact within early data assemblies from The Cancer Genome Atlas (TCGA) [8]. For over a decade, baseline discovery studies heavily relied on TCGA’s designated “normal” controls to establish baseline expression metrics for healthy organs. However, systematic pan-cancer transcriptomic analyses, such as the benchmark evaluation by Aran et al. [9], have since demonstrated that histologically normal tissue adjacent to the tumor (NAT) does not equate to genuine biological normalcy. Instead, NAT captures a highly active, unique intermediate molecular state situated between a truly non-diseased baseline and frank malignancy.

In the context of the highly infiltrative microenvironment characteristic of PDAC, using NAT controls introduces significant confounding variables. One variable is the pro-inflammatory signaling fields. Malignant cells continuously shed paracrine stimuli, cytokine networks (such as TNF-α), and growth factors into the surrounding tissue margins [9]. This sustained chemical stress activates an intense inflammatory response, remodeling the adjacent vascular endothelium, extracellular matrix, and stroma. Another variable that altered results of the ‘normal’ TCGA tissues involves stromal cross-contamination. Because healthy exocrine pancreatic tissue lacks baseline *CCKBR* expression, its apparent expression is strictly restricted to rare neuroendocrine islet cells. However, within the active stromal margins captured in NAT samples, *CCKBR* becomes robustly upregulated during early localized desmoplasia and pre-neoplastic field effects.

Consequently, comparing primary tumors against adjacent NAT controls artificially minimizes the apparent expression gap. When *CCKBR* levels in the tumor were evaluated against these falsely elevated TCGA “normal” baselines, the true differential expression was mathematically hidden. Correcting this systematic artifact requires a complete data separation strategy: completely discarding cross-contaminated NAT profiles, and cross-referencing tumor sequences with uncompromised, non-tumor-bearing pancreatic specimens from repositories like the Genotype-Tissue Expression (GTEx) Project [10]. By isolating these healthy cohorts, the current work reveals a substantial, early-stage binary transcriptomic switch (*p* = 1 × 10^−60^) that marks true pancreatic carcinogenesis. By establishing the clinical significance of *CCKBR* expression, this study introduces new opportunities to utilize the CCKBR protein for early diagnostic imaging and therapeutic targeting.

## 2. Materials and Methods

### 2.1. Data Source and Patient Cohort Stratification

To evaluate the transcriptional landscape of *CCKBR* in human pancreatic tissue, uniformly processed bulk RNA-sequencing (RNA-seq) datasets were retrieved from the Genotype-Tissue Expression (GTEx) portal [10] and The Cancer Genome Atlas (TCGA) repository [8]. To prevent confounding structural or batch biases introduced by adjacent non-tumor samples, all normal tissue controls originating from the TCGA-PAAD project were omitted. The final benchmark cohorts comprised the following: healthy baseline tissue of post-mortem normal human pancreas specimens from the GTEx repository (*N* = 328) and malignant tissue: primary pancreatic adenocarcinoma samples from the TCGA-PAAD repository (*N* = 178). The raw data for each of these cohorts is available in Supplementary Data Table S1.

### 2.2. RNA-Seq Processing and Abundance Normalization

Raw sequencing files from both consortia were re-aligned to the human reference genome (GRCh38/GENCODE v23) using the Toil pipeline to minimize cross-study technical batch effects [11]. Transcript abundances were quantified and normalized as Transcripts Per Million (TPM) to adjust for sequencing depth and gene length variations [12]. To stabilize variance and account for the highly skewed distribution of low-abundance transcripts, expression metrics were linearized using the standard logarithmic transformation (log_2_TPM + 1).

### 2.3. Characterization of the SNP in the 4th Intron of the CCKBR Gene

In a prior study, de-identified tissues were obtained from the tumor banks of three Cancer centers (Penn State Hershey, University of Pittsburgh, and MD Anderson). A de-identified normal human pancreas that was procured but not used for transplantation was obtained from the Gift of Life program. DNA was isolated from pancreas tissues using the DNeasy kit (Qiagen). Two hundred nanograms of each resultant DNA sample was then amplified by CCKBR primers upstream (5′-GCC CTA CCC CGT GTA CAC TGT CGT G-3′) and downstream (5′-CGG TGT GCA CCC GGG CCA TCA AAG GCG C-3′) using the Phusion High-Fidelity PCR Master Mix with GC buffer (New England BioLabs) to produce a 1049 bp product. The PCR products were purified (QIAquick PCR Purification Kit, Qiagen) and sequenced using a 5′-GCA CTA TAA ACT GGC AAC CAA CAC AG-3′primer with an ABI 3130XL Capillary sequencer and confirmed using a TaqMan SNP Genotyping Assay (C__22273290_10). Survival was analyzed by Kaplan–Meier analysis comparing the wild-type CC genotype to the SNP variant genotypes (AA and AC).

### 2.4. Calculation of Meta-Analysis for Splice Variant Kaplan–Meier Survival Data

To conduct a pooled individual patient data (IPD) meta-analysis, individual survival times, event indicators, and censoring information were reconstructed from the published high-resolution Kaplan–Meier curves of the included studies utilizing the iterative numerical algorithm described by Guyot et al. [13]. Digitized coordinates of the survival curves and total numbers of patients at risk were processed to recover the underlying patient-level data matrix with high fidelity. Prior to matrix unification, baseline study-level heterogeneity across the independent source cohorts was evaluated using Cochran’s Q test and the I^2^ statistic to quantify historical inter-study variance.

To guarantee cohort independence and eliminate overlapping participants between early institutional registries, a cross-table audit was performed, resulting in the successful identification and exclusion of 24 duplicate patient records (2 A/A, 7 C/A, and 15 C/C). Survival trajectories were evaluated under a dominant genetic model over a 60-month follow-up window, pooling homozygous and heterozygous variant carriers into a single cohort (A Allele Carriers, *N* = 330) against wild-type controls (C/C Wild-type, *N* = 750). Cumulative overall survival curves were constructed using the Kaplan–Meier method, and statistical significance was determined using a two-sided log-rank test using the survival package in R version 3.8-12.

### 2.5. Validation of CCKBR Protein Expression in Human Precancerous PanINs

CCKBR immunohistochemistry was performed on a human pancreas tissue microarray (US Biomax, Cat# BIC14011b; Rockville, MD, USA) [14]. This microarray contained 48 unstained cores of fresh frozen paraffin-embedded human pancreas tissues from both normal pancreases obtained at autopsy and from subjects with various grades of precancerous pancreatic intraepithelial neoplasia (PanINs). The tumor array slide was washed in 1× PBS 3× for 2 min and then blocked using the anti-goat HRP-DAB IHC Detection kit (Novus Biologicals, Cat# cts008-NOV; Littleton, CO, USA). The slide was then incubated with the primary antibody CCKBR (Abcam, Cat# Ab77077; Cambridge, UK) at 1:200 titer in PBS overnight at 4 °C. After rinsing, the slide was incubated with 1–3 drops of Biotinylated Secondary Antibody (Vial A, Novus) for 60 min followed by 1–3 drops of HSS-HRP (Vial B, Novus) for 30 min and washed, and DAB Chromogen was added for 3 min.

### 2.6. In Vivo Imaging of PanINs with CCKBR-Targeted Nanoparticles in Mice

To minimize unnecessary animal duplication and adhere to ethical research standards, the findings in this current nanoparticle work are integrated with previously published and unpublished data from a historically published experiment [14]. All animal studies were performed in an ethical fashion under a protocol approved by the Georgetown University IACUC. Five-to-ten-month-old p48-Cre/LSL-Kras^G12D^ (KC) mice were used in this investigation (Total *N* = 34 mice). Of these mice, *N* = 12 KC mice, ages 5, 6, 7, 8, and 10 mos, were injected with targeted fluorescent NPs; *N* = 12 KC mice (age matched) were injected with untargeted NPs; *N* = 5 KC mice were injected with a diluent; and *N* = 5 wild-type C57BL/6 mice were injected with targeted NPs.

PanINs begin to develop by 3 months in this transgenic model, and PanIN-3 lesions are histologically identified by 4–6 months. For imaging purposes, the polyplex nanoparticle (NP) was modified using two separate non-specific oligonucleotides complexed with fluorophores: Alexa Fluor 647 and Alexa Fluor 488. The Alexa Fluor 647-tagged NP was selected for its fluorescent properties in the far-red wavelength range that allowed for in vivo imaging of mutant Kras mice with a Lumina III in Vivo Optical Imaging System (IVIS). The Alexa Fluor 488-tagged NP was used for localization of the NP by immunohistochemistry. Mice were placed on an alfalfa-free chow (ENVIGO, cat# TD.97184; Indianapolis, IN, USA), and the fur was shaved to optimize imaging. The CCKBR-targeted NPs or untargeted NPs (480 nM) were injected intraperitoneally in a volume of 100 µL, and the mice were imaged over 24 h, with peak bioluminescence detected over the pancreas area at 5 h of the CCKBR-targeted NPs. The major organs and pancreas were excised after 5 h and imaged in the IVIS ex vivo to demonstrate the specificity of the NP for the CCKBR-positive lesions in the pancreas. Controls included KC mice injected with untargeted NPs or a diluent, and wild-type mice injected with the CCKBR-targeted NPs.

In order to confirm that the fluorescent-labeled targeted polyplex NPs localized to the CCKBR expressing pancreatic PanIN lesions, the pancreata were fixed and paraffin-embedded for histology with hemoxylin and eosin staining and immunohistochemistry. Tissue sections were reacted with an antibody to Alexa Fluor 488 (1:400; Invitrogen, Cat# A-11094; Waltham, MA, USA) to confirm the uptake of the NPs into PanIN lesions. Slides from the Alexa Fluor 488 immunohistochemistry were scanned on an Aperio ScanScope XT (Leica) and intensity of immunofluorescence calculated using the QuPath software (Version v0.2.3) from *N* = 10 of each PanIN grade (1, 2, and 3) analyzed in slides from (*N* = 3) 5-month-old mouse pancreas. The corresponding mouse pancreas was stained with H&E to reveal the characteristic PanIN histology. Detailed methods for the earlier experiments are described in a previous publication [14].

### 2.7. Statistical Assessment and Data Visualization

For the *CCKBR* transcriptomic analysis, individual sample distributions were visualized using a scatter plot configuration coupled with a custom horizontal point-jittering algorithm to preserve data-point density granularity. Statistical variations in *CCKBR* expression distribution between the normal (GTEx) and tumor (TCGA) cohorts were assessed using a two-sided, non-parametric Wilcoxon rank-sum test (Mann–Whitney U test), as data distributions violated normality assumptions. The exact probability value is reported down to the analytical software threshold (*p* = 1 × 10^−60^). All statistical tests were two-tailed, and an alpha level of 0.05 was considered statistically significant.

For prognostic evaluation, overall survival (OS) curves were estimated using the Kaplan–Meier method based on the primary TCGA-PAAD cohort (*N* = 178). Patients were stratified into two equal arms (*n* = 89 each) representing high or low CCKBR expression using a median split configuration. Statistical differences between the survival distributions of the high-expression and low-expression groups were evaluated using a two-sided log-rank (Mantel–Cox) test. To estimate the relative mortality risk, a univariate Cox proportional hazards regression model was employed to calculate the hazard ratio (HR) along with its corresponding 95% confidence interval (CI).

To evaluate the diagnostic utility of *CCKBR* gene expression levels in distinguishing malignant tissue from healthy baseline controls, a receiver operating characteristic (ROC) curve analysis was implemented. The overall discriminatory performance was quantified by calculating the area under the ROC curve (AUC), with the statistical significance of the classifier evaluated against a random classifier (AUC = 0.50).

To analyze and visualize *CCKBR* gene expression across different pathologic stages of pancreatic cancer, raw transcriptomic data in Transcripts Per Million (TPM) along with patient staging metadata were retrieved from the TCGA database and combined with normal pancreas tissue baseline data from the GTEx database. Pathologic stages were stratified into Stage 1 (*N* = 21), Stage 2 (*N* = 146), and a combined Stage 3/4 group (*N* = 11). Computational workflows, descriptive cohort statistics, and plotting configurations were developed in Python (v3.10) using the Pandas and SciPy libraries for data manipulation and correlation metrics, and Matplotlib (version 3.11.2) combined with Seaborn for graphic rendering.

For the splice-variant *CCKBR* alteration, the cumulative survival differences between the wild-type and risk-allele cohorts of the CCKBR were statistically scrutinized using a two-sided log-rank (Mantel–Cox) test. To quantify overall relative mortality indicators across the multi-center populations while adjusting for local study design variants, both fixed-effect and random-effect Cox proportional hazard regression models were run in parallel. Inter-study statistical heterogeneity was explicitly evaluated using Cochrane’s *Q* test and quantified via the *I*^2^ index. Due to the fundamental clinical divergence observed between the North American multi-center datasets and the Central European validation cohorts, a pre-planned secondary subgroup analysis stratified by continental tracking demographics was deployed to evaluate population-specific hazard variations independently. All pooled statistical calculations, hazard distributions, and aggregate confidence boundaries were computed using standard packages in R.

The key resources from on-line websites are listed below in Table 1.

## 3. Results

### 3.1. Analysis of CCKBR Differential Expression in Pancreatic Cancer

**Figure 1:**
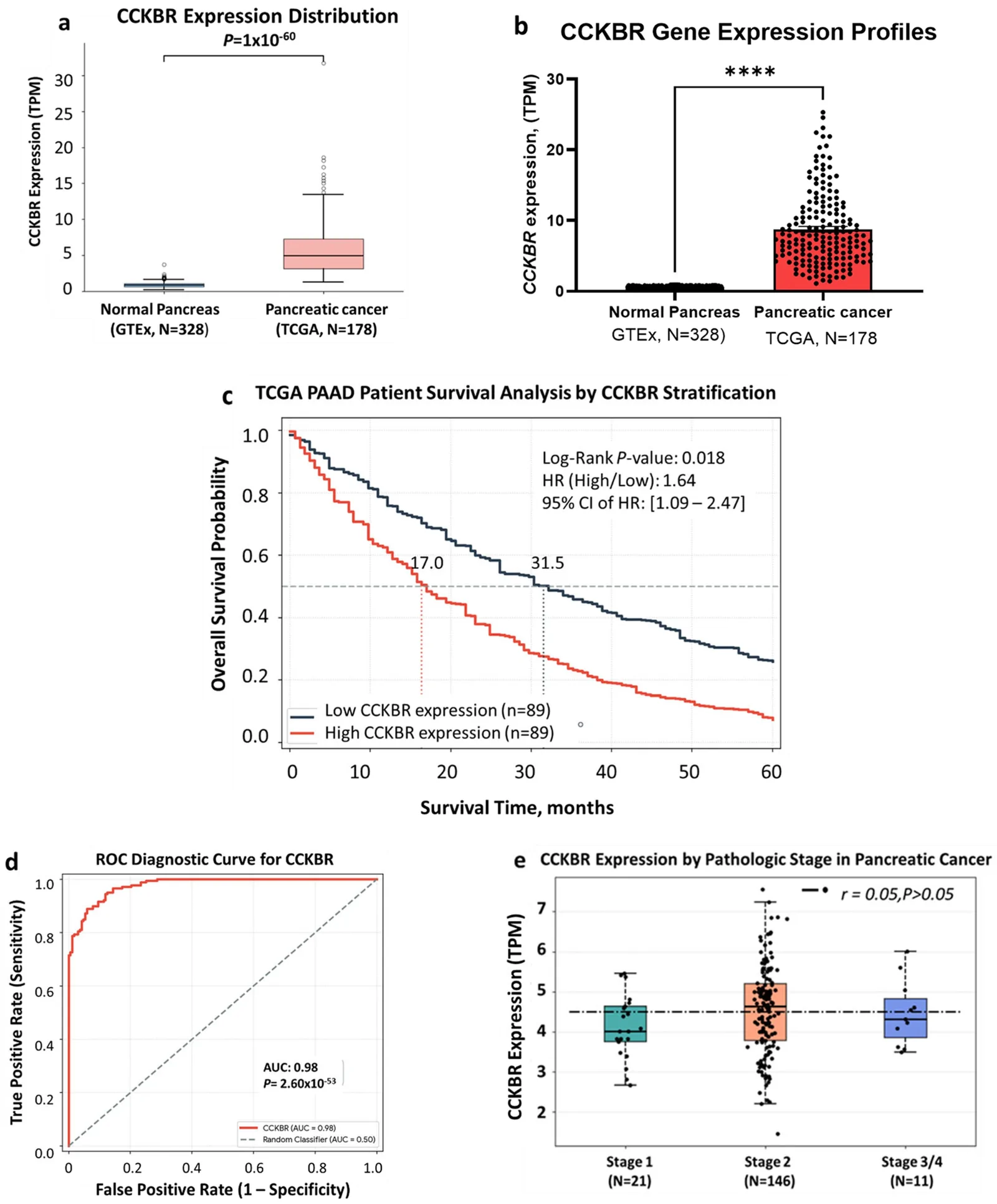
Differential expression of *CCKBR* in normal and malignant pancreatic tissues. (**a**) Box plots illustrating the transcriptional levels (Transcripts Per Million, TPM) of the cholecystokinin B receptor (*CCKBR*) in normal pancreas tissues (*N* = 328, blue) derived from the Genotype-Tissue Expression (GTEx) database compared with pancreatic cancer tissue samples (*N* = 178, light red) obtained from The Cancer Genome Atlas (TCGA). Center lines indicate median values, the bounds of the boxes represent the interquartile range (IQR), and the whiskers denote 1.5 times the IQR. Open circles represent statistical outliers. Statistical analysis was performed using a two-tailed Wilcoxon rank-sum test (Mann–Whitney U test) on log-transformed data [(log2TPM + 1)], demonstrating a highly significant upregulation of *CCKBR* expression in malignant tissues compared to normal controls (*p* = 1 × 10^−60^). (**b**) Scatter plot and distribution layout illustrating *CCKBR* mRNA expression levels (quantified in TPM) between healthy control and malignant cohorts. **** *p* < 0.0001 using unpaired Welch’s *t*-test. (**c**) Kaplan–Meier overall survival analysis stratified by *CCKBR* gene expression in TCGA database for pancreatic adenocarcinoma (PAAD). Patient data were stratified into two distinct groups using a median expression cutoff: High *CCKBR* Expression (*n* = 89, red line) and Low *CCKBR* Expression (*n* = 89, dark grey line). Dotted lines designate the specific coordinates for median survival time points, showing a marked reduction in clinical survival from 31.5 months in the low-expression group down to 17.0 months in patients overexpressing *CCKBR*. (**d**) A receiver operating characteristic (ROC) curve analysis shows how effectively *CCKBR* expression functions as a diagnostic biomarker to distinguish pancreatic cancer (TCGA-PAAD) from true healthy pancreatic tissue (GTEx). The biomarker achieved an AUC of 0.98 (95% CI: 0.96 to 1.00). DeLong’s non-parametric method was utilized to calculate the variance and compare the curve against the random performance line (AUC = 0.50). The resulting Z-score was mapped to a standard normal distribution curve to extract the two-tailed significance level (*p*-value = 2.6 × 10^−53^). (**e**) Distribution of *CCKBR* gene expression across pathologic stages of PDAC stage-stratified box plot mapping *CCKBR* mRNA expression levels, quantified as log_2_(TPM + 1) values, across progressive clinical pathology within the TCGA-PAAD cohort. Samples are partitioned into Stage 1 (*N* = 21, teal), Stage 2 (*N* = 146, coral), and a combined Stage 3/4 cohort (*N* = 11, blue). Horizontal lines within the colored boxes represent median values, box boundaries denote the interquartile range (IQR), and vertical whiskers extend to 1.5 times the IQR. Individual patient samples are overlaid as solid black jittered points to clearly show data density and patient-to-patient heterogeneity.

To evaluate the potential role of the CCKBR in pancreatic malignancy, its transcriptional profile was analyzed across normal and neoplastic pancreatic tissues. RNA-sequencing data from the GTEx database (*N* = 328) normal pancreas samples were compared against pancreatic cancer samples from the TCGA database (*N* = 178). Figure 1a shows that CCKBR expression is significantly elevated in pancreatic cancer tissue compared to normal pancreatic tissue. The differential expression is statistically significant, with a calculated *p*-value of 1 × 10^−60^. Individual data-points are plotted (Figure 1b) to visualize data density, demonstrating a tightly restricted baseline expression in normal tissue compared to high heterogeneity and prominent upregulation in tumor samples.

Overall survival status was calculated by the Kaplan–Meier survival curve to compare low versus higher CCKBR expression in the TCGA-PAAD cancer cohort (Figure 1c). Patients with lower CCKBR expression demonstrated a clinical advantage, achieving a median overall survival of 31.5 months (HR, high/low = 1.64; 95%CI: 1.09–2.47). Those in the high-CCKBR-expression cohort drop sharply to a median overall survival of only 17.0 months. Consistent with the surgical sample acquisition criteria of the TCGA-PAAD cohort, which excludes the majority of patients presenting with unresectable, advanced-stage disease at initial diagnosis, the median overall survival for both the high- and low-CCKBR-expression groups was prolonged relative to general public health registries.

To evaluate how effectively CCKBR expression functions as a diagnostic biomarker to distinguish pancreatic cancer (TCGA-PAAD) from true healthy pancreatic tissue (GTEx), a receiver operating characteristic (ROC) curve analysis was performed. ROC analysis (Figure 1d) demonstrated strong diagnostic accuracy for CCKBR, significantly outperforming random chance (*p* < 0.0001). The diagnostic capability of CCKBR separating pancreatic tumor samples from clean pancreatic controls is highly statistically significant, achieving an AUC of 0.98 (95% CI: 0.96 to 1.00). Because the entire 95% confidence interval range sits comfortably above the random performance line of 0.50, the metric confirms high predictive precision. Even when factoring in data variance, sample replacement errors, and outlier shifts across thousands of random patient cohorts, the classification accuracy remains exceptionally strong.

To evaluate whether CCKBR expression escalates as a function of tumor progression, the primary PDAC samples were stratified by clinical pathology (Figure 1e). Transcript abundance is already markedly elevated during early localized disease, with Stage I tumors (*N* = 21) exhibiting a high median expression of approximately 4.1 log_2_(TPM + 1). This robust baseline expression remains firmly sustained into Stage 2 tumors (*N* = 146), which represent the predominant surgical subpopulation and feature a steady median of 4.66 log_2_(TPM + 1). In advanced and metastatic settings, represented by the combined Stage 3/4 cohort (*N* = 11), CCKBR expression is fully maintained at a median of 4.29 log_2_(TPM + 1). These findings demonstrate that CCKBR is markedly overexpressed in a substantial proportion of PDAC, suggesting its potential utility as a diagnostic biomarker or a therapeutic target.

### 3.2. Alternative Splice Variant of the CCKBR

**Figure 2:**
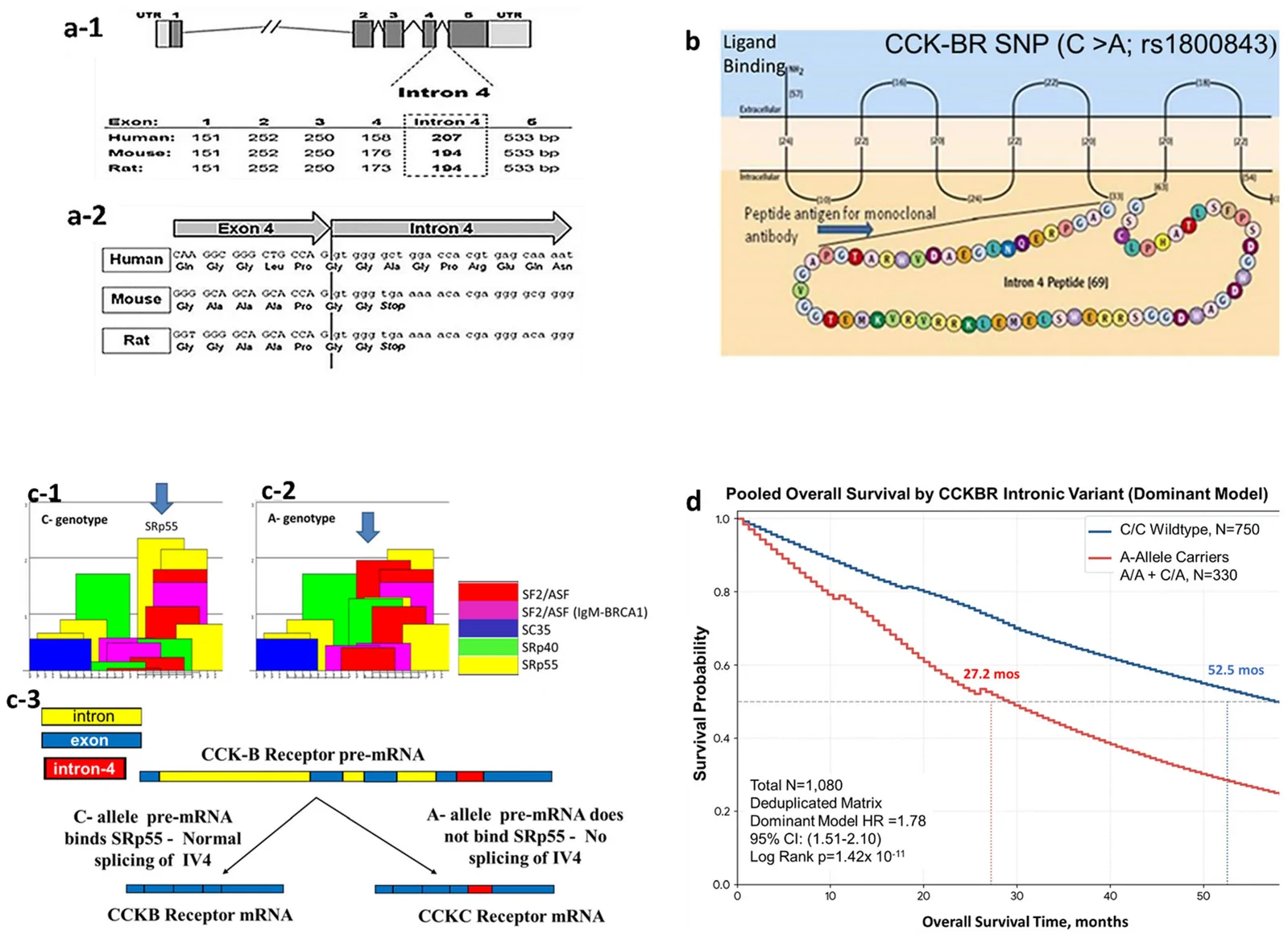
Mis-splicing of the CCKBR due to an SNP, rs1800843. (**a-1**) Diagram of the nucleotide number for the 4th intron comparing the human *CCKBR* receptor to that of the mouse and rat. (**a-2**) Nucleotide sequences of human *CCKBR* shows an open reading frame in the 4th intron, whereas the murine *Cckbr* receptor has a stop codon early in the 4th intron. (**b**) Diagram showing the mis-splicing of intron 4 of the CCKBR gene leads to a 69-amino-acid insertion in the 3rd intracellular loop. A model of the CCK-BR SNP genotype splicing interactions. (**c-1**) Using a splicing factor binding site database, several SR proteins are identified that are involved with splicing intron 4 in the presence of the CC genotype. (**c-2**) Shows the SR proteins and the absence of the prominent SRp55 protein binding when the A allele is expressed. The rs1800843 SNP falls within a predicted binding site for the splicing factor SRp55. (**c-3**) In the presence of the C- allele pre-mRNA binds SRp55 resulting in normal splicing of intron 4 (IV4). In the presence of the A allele, pre-mRNA does not bind SRp55 and mis-splicing of the 4th intron of the *CCKBR* occurs leading to the phenotype of the CCK-C receptor. (**d**) Global consolidated analysis of overall survival by *CCKBR* intronic SNP genotype. Kaplan-Meier survival curves illustrate overall survival time (in months) over a 60-month follow-up window for a meta-cohort of 1080 unique patients evaluated under a dominant genetic model. Patients with the homozygous wild-type genotype (C/C; *n* = 750, blue line) exhibited a significantly prolonged median overall survival of 52.5 months compared to variant carriers (A-Allele carriers [A/A + C/A], *n* = 330, red line), who demonstrated a median overall survival of 27.2 months (log-rank; *p* = 1.42 × 10^−11^). The consolidated analysis integrates individual patient data from three cohorts after the exclusion of 24 overlapping records, (Cleaned, *n* = 27; hazard ratio [HR] = 3.12, 95% CI: 1.65–5.90) [16], (*n* = 931; HR = 1.83, 95% CI: 1.52–2.21) [17], and (*n* = 122; HR = 1.04, 95% CI: 0.72–1.51) [18], which showed a neutral, non-significant prognostic effect when analyzed in isolation. The overall pooled fixed-effect model yielded an aggregate (HR = 1.78, 95%CI: 1.51–2.10). Dashed horizontal and vertical lines indicate the median survival thresholds for each genetic cohort. Figure 2(c-1–c-3) reproduced with permission [17].

**Figure 3:**
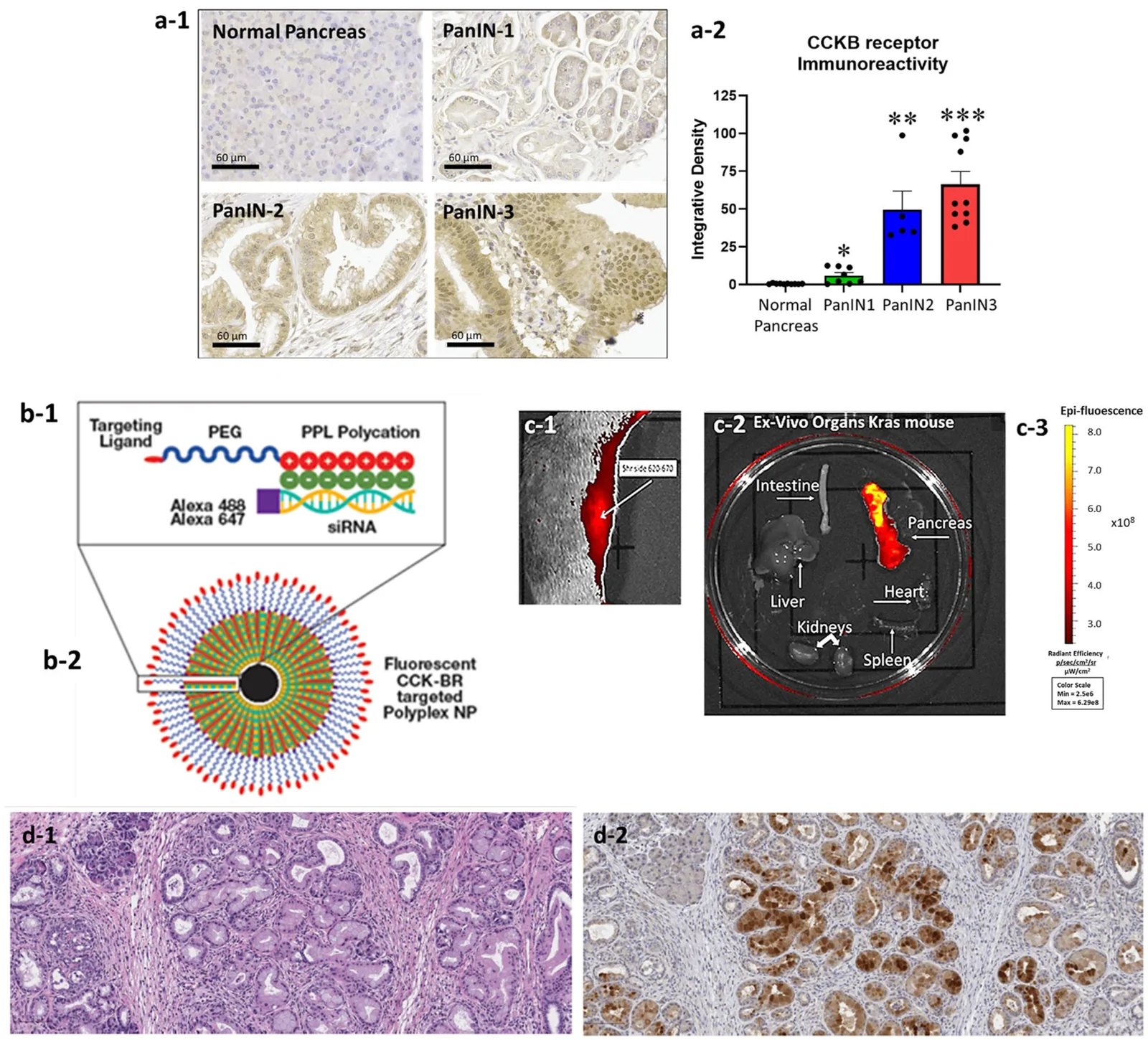
CCKBR overexpression in PanINs and PDAC provides a unique target for early diagnosis and targeted therapy. (**a-1**) Representative images from a human pancreas tissue array showing normal human pancreas tissue and human pancreas tissues with various PanIN-1, PanIN-2, and PanIN-3 stained with a CCKBR antibody. (**a-2**) Quantitative analysis of all the images from the human pancreas tissue array shows absence of the CCKBR in normal pancreas and increased CCKBR immunoreactivity with increasing grade of PanIN lesions. Results are significantly different from the normal pancreas tissues * *p* < 0.05; ** *p* < 0.01; *** *p* < 0.005. (**b-1**) Diagram of the polyplex nanoparticle backbone constructed with a polyethylene glycol-block-poly(L-lysine)(SH-PEG-PLL) polymer connected to a functionalized receptor-binding 10-amino-acid ligand using a thiol-maleimide coupling reaction. (**b-2**) When reacted with negatively charged siRNA, it forms a polyplex micelle with the CCK-BR-targeting moiety on the outside. (**c-1**) In vivo imaging of p48-Cre/LSL-Kras^G12D^ (KC) mouse post-injection of CCKBR-targeted Alexa 647 polyplex nanoparticle shows uptake in the pancreas. (**c-2**) Ex vivo fluorescent imaging of major organs removed post-injection in KC mice with CCKBR-targeted nanoparticle and (**c-3**) scale bar for magnitude of fluorescence radiant efficiency. (**d-1**) H&E histologic section of the 10-month-old mouse pancreas reveals evidence of high-grade PanIN lesions and fibrosis of the pancreas microenvironment (bar = 100 µm). (**d-2**) The same pancreas section probed with anti-Alexa Fluor 488 antibody shows marked immunoreactivity, consistent with the CCKBR-targeted polyplex micelle nanoparticle localization in advanced PanIN lesions. (Bar = 100 µm.) Figure 3(a-1,a-2,c-2) were previously published in *Biomolecules.* **2021**, 11, 1766 [14].

The identification of reliable prognostic and diagnostic molecular biomarkers remains a critical challenge in improving the dismal survival rates associated with PDAC. We identified a splice variant of the *CCKBR* in the 4th intron [15]. This single-nucleotide polymorphism (SNP) was not originally identified in the broad GWAS because it is intronic rather than exonic. Also, this splice variant occurs only in humans and not in mice because the 4th intron of the *CCKBR* gene in humans contains 207 nucleotides, which (divided by 3 bases per codon) yields a peptide of 69 amino acids in length. Both the mouse and rat *Cckbr* genes contain 194 nucleotides (not divisible by 3). The CCKBR structure and size of the exons and 4th intron are shown in Figure 2(a-1). The human *CCKBR* gene has an open reading frame in the 4th intron, allowing for its full translation, unlike the murine intron, which contains a stop codon early in the 4th intron (Figure 2(a-2)). The alternative splicing of the 4th intron of the *CCKBR* gene leads to the retention of intron 4 (IVS4), creating a constitutively active, pro-proliferative receptor isoform (or CCKBR Isoform 2) also known as the CCK-C receptor (Figure 2b) [15]. Genotyping the human PDAC tissues identified a single-nucleotide polymorphism (SNP; rs1800843, c.811+32C>A) within this intronic sequence as the etiology of the mis-splicing. This variation structurally alters the consensus binding domain for the SRp55 splicing factor and independently links to highly aggressive tumor phenotypes (Figure 2(c-1–c-3)). Using a splicing factor binding site database several SR proteins were identified that are involved with splicing intron 4 in the presence of the CC genotype (Figure 2(c-1)). In the presence of the wild-type C-allele pre-mRNA binds SRp55, resulting in normal splicing of intron 4 (IV4) (Figure 2(c-1)). When the SNP A allele is expressed, the SR proteins show an absence of the prominent SRp55 protein binding (Figure 2(c-2)). The rs1800843 SNP falls within a predicted binding site for the splicing factor SRp55. In the presence of the A allele, pre-mRNA does not bind SRp55, and mis-spicing of the 4th intron of the *CCKBR* occurs, leading to the phenotype of the CCK-C receptor (Figure 2(c-3)).

A pilot cohort by Smith et al. [16] linked the presence of minor A alleles to a nearly four-fold elevation in mortality risk (hazard ratio [HR] = 3.63), a finding heavily reinforced by a subsequent large-scale, multi-center validation trial encompassing 931 American patients [17], which established a highly robust statistical correlation (*p* = 3.11 × 10^−11^) after rigorous staging adjustments. However, this oncogenic paradigm faced distinct geographic divergence. A subsequent European validation trial conducted by the Hungarian Pancreatic Study Group [18] in a cohort of 122 Hungarian PDAC patients observed zero functional survival deviation or splicing differences between standard wild-type (C/C) genotypes and minor-allele risk carriers (A/C or A/A). These conflicting trans-Atlantic outcomes highlight a complex layer of genetic penetration. They imply that the prognostic efficacy of the rs1800843 variant is not universally absolute but rather modulated by demographic-specific driver mutations, distinct genetic backgrounds, or regional variations in background spliceosome efficiency.

To resolve the conflicting prognostic values reported across isolated geographic populations, a unified meta-analysis was executed by pooling raw patient clinical data and genotypic indexes from all peer-reviewed studies tracking the *CCKBR* intronic rs1800843 SNP in human pancreatic adenocarcinoma. The consolidated master dataset comprised the three independent historical cohorts spanning from 2012 to 2016 [16,17,18], aggregating a cumulative population of *N* = 1080 patients. Baseline assessment of these underlying study populations revealed significant study-level heterogeneity (Cochran’s Q = 10.85, df = 2, *p* = 0.0044; I^2^ = 81.6%). Patient clinical profiles were standardized, and individuals were cleanly partitioned into two binary prognostic strata: a wild-type group consisting exclusively of homozygous C/C genotype individuals, and a minor allele carrier group pooling heterozygous A/C and homozygous A/A variant individuals to preserve original analytical configurations across all underlying source methodologies. Time-to-event survival trajectories comparing A-allele carriers against wild-type controls within the global consolidated cohort were modeled using the non-parametric Kaplan–Meier method over an extended 60-month follow-up window (Figure 2d). Patients with the wild-type *CCKBR* (C/C genotype, blue line) exhibited a significantly prolonged median overall survival of 52.5 months compared to minor allele carriers (A/C or A/A genotypes, red line), who demonstrated a median overall survival of 27.2 months (composite system significance: *p* = 1.42 × 10^−11^). Study-specific hazard ratios, the unified individual patient data pooled model, and cohort heterogeneity statistics for CCKBR intronic variant expression are found in Supplementary Table S2.

According to the National Center for Biotechnology Information (NCBI) dbSNP database, this single-nucleotide variation primarily involves a cytosine-to-adenine (C>A) substitution. Aggregate data from the Allele Frequency Aggregator (ALFA) project indicates a global reference (C) allele frequency of 81.9% and an alternate (A) allele frequency of 18.1%, though the minor allele frequency varies across populations, falling to approximately 3.2% in Asian cohorts. While this germline variant maintains a well-defined baseline in healthy populations, its prevalence rises sharply to about 40% among patients diagnosed with PDAC. Functionally, this C>A transversion modifies standard molecular processing to drive the alternative splicing of *CCKBR*, yielding a constitutively active receptor isoform (CCK-CR) that stimulates autocrine growth loops and accelerates tumor cell proliferation.

### 3.3. Clinical Implications of the CCKBR for Diagnostics

One reason contributing to the poor outcome of this malignancy is the inability to diagnose PDAC in early or precancerous stages [19]. Current radiographic imaging tools such as computerized tomography (CT) and magnetic resonance imaging (MRI) lack sensitivity [20] and are limited to detecting tumors greater than 2 cm in size [21]. Endoscopic ultrasound is another approach that has been used in clinics to evaluate pancreatic cysts or intraductal papillary mucinous neoplasms (IPMNs) for PDAC [22]. However, only about 15% of pancreatic cancers arise from cysts; the majority (85%) of pancreatic cancers develops from a microscopic precursor lesion called pancreatic intraepithelial neoplasia (PanINs). Unfortunately, PanINs are not identified by standard radiographic imaging or endoscopic techniques.

To determine whether CCKBR is expressed in precancerous human PanIN lesions, a human pancreas PanIN tissue array with various PanIN grades was reacted with a CCKBR antibody and integrative density of the staining measured [14]. CCKBR protein immunoreactivity is shown from the human pancreas array with varying PanIN grades (Figure 3(a-1)). No staining was found in the normal human pancreas, a result consistent with the lack of gene expression in the normal tissue from the GTEx data. However, the immunoreactivity for the CCKBR protein increased with PanIN grade (Figure 3(a-2)). A polyplex nanoparticle (NP) was developed that selectively binds to the CCKBR. This NP was modified with a fluorophore (Alexa647) for imaging purposes in vivo (Figure 3(b-1,b-2)). Additionally, the NP was designed to carry a payload of an siRNA-labeled marker with Alexa 488 for immunohistochemical localization. With the detection of CCKBR on PanIN lesions, the feasibility of using this cell surface receptor as a critical biomarker for early-stage PDAC diagnosis with imaging was explored [14]. The efficacy of CCKBR-targeted polyplex nanoparticles was examined using p48-Cre/LSL-Kras^G12D^ (KC) mice that develop precancerous PanIN lesions of increasing grades over time. After injection of the fluorescent nanoparticles, to KC mice, epi-fluorescence was localized over the pancreas using an in vivo imaging system (IVIS) (Figure 3(c-1)). The pancreas and major organs were excised and imaged ex vivo in the imaging system, revealing radiant efficiency only in the excised pancreas and not in other organs (Figure 3(c-2)). A scale bar for bioluminescence is displayed in Figure 3(c-3). Histologic evaluation of the pancreas with hematoxylin and eosin staining confirmed the presence of PanIN lesions (Figure 3(d-1)), and localization of nanoparticle uptake into the PanINs, and not the adjacent normal pancreatic acinar cells, was confirmed by immunohistochemistry with the Alexa 488 antibody (Figure 3(d-2)). Since PanINs are not detected with routine imaging, using the CCKBR receptor as a target to enhance radiographic imaging could lead to early detection of precancerous lesions. These results suggest that CCKBR represents a high-potential target for early diagnosis, as its overexpression in pre-invasive PanIN lesions enables identification via targeted fluorescent nanoparticles. In addition to serving as a diagnostic tool, we have modified the CCKBR-targeted nanoparticles to deliver siRNA payloads to specifically knockdown mutant Kras [23] or other driver genes like gastrin [24], rendering the nanoparticle as a tool for both diagnosis and treatment—or a theranostic agent.

## 4. Discussion

One reason contributing to the poor outcome of PDAC is the challenge of detecting early-stage or precancerous lesions, such as pancreatic intraepithelial neoplasia (PanINs) using standard radiographic imaging [20]. Additionally, the dense, desmoplastic stroma acts as a physical barrier that restricts drug delivery and immune cell infiltration, leading to poor therapeutic efficacy [25]. In the current investigation, the significance of the *CCKBR* transcriptional profile was analyzed across normal and neoplastic pancreatic tissues, demonstrating tightly restricted baseline expression in normal tissue versus prominent upregulation in tumor samples. The observed median overall survival times in our stratified cohorts (17.0 and 31.5 months) are notably longer than typical historical benchmarks for pancreatic adenocarcinoma; this discrepancy is attributable to an inherent selection bias within the TCGA-PAAD repository, which predominantly aggregates tissue samples from patients who successfully underwent surgical resection for localized or regional disease. The persistent, early-stage upregulation in pathology specimens that was demonstrated highlights the clinical significance of the receptor, and the Kaplan–Meier survival curves revealed that high *CCKBR* expression correlates with a sharp drop in median overall survival compared to patients with low expression.

We acknowledge that the highly discriminative diagnostic performance of *CCKBR* captured by our ROC analysis (AUC = 0.98) relies on a cross-database comparison between bulk surgical tumor specimens (TCGAs) and post-mortem healthy pancreas controls (GTEx). While this framework provides excellent statistical power for initial biological validation, it does not fully replicate a real-world clinical diagnostic setting. Post-mortem tissue acquisition introduces unique warm ischemic intervals and tissue degradation profiles that differ from living clinical specimens. Furthermore, a practical clinical biomarker must distinguish malignancy from overlapping inflammatory conditions, such as chronic pancreatitis or benign cystic lesions. Consequently, prospective validation using uniform, living patient cohorts and biopsy-derived tissue matrices is required to definitively establish the diagnostic precision of CCKBR in clinical practice. Unfortunately, subjects with a healthy or ‘normal’ pancreas do not undergo routine biopsy.

A key consideration when evaluating bulk tissue RNA-sequencing matrices, such as the TCGA-PAAD dataset, is that the cellular architecture represents a blended transcriptomic signal from malignant cells and dense microenvironmental stroma. However, our independent in vitro characterization of isolated human pancreatic cancer cells confirms a definitive epithelial origin, showing robust baseline CCKBR expression in contamination-free malignant cell lines [26,27]. Functional activation of epithelial CCKBR triggers downstream signaling cascades via the AKT and Kras pathways to directly stimulate cancer cell proliferation, while in vivo knockout models or pharmacologic blockade inhibit tumor growth [28,29,30]. The malignant epithelial cells themselves engage in potent paracrine cross-talk with neighboring pancreatic stellate cells (PSCs) and local fibroblasts. Upon activation via these reciprocal epithelial–stromal networks, the neighboring PSCs orchestrate the massive collagen deposition and desmoplastic fibrosis characteristic of the PDAC ecosystem. Interruption of the epithelial–stroma communication is altered with blockade or downregulation of CCKBR [30,31]. Pancreatic cancer cells also cross communicate with neighboring immune cells to render the tumor microenvironment immunosuppressive, such as inducing polarization of macrophages to the M2 phenotype [32]. Thus, the prominent CCKBR signature captured in bulk TCGA data reflects a primary malignant epithelial driver that actively coordinates the surrounding fibrotic microenvironment. Nonetheless, prospective single-cell RNA sequencing (scRNA-seq) or spatial transcriptomics will remain valuable future directions to further map these discrete cellular topographies in vivo.

The transcriptomic and genetic findings were validated at the protein level, demonstrating that CCKBR expression is significantly upregulated in human PanIN lesions in a grade-dependent manner while remaining completely absent in normal tissue [14]. In a p48-Cre/LSL-Kras^G12D^ mouse model, CCKBR-targeted polyplex nanoparticles were specifically directed to pre-invasive PanIN lesions and visualized via in vivo and ex vivo fluorescence imaging. This targetable approach serves a dual role as a theranostic agent capable of delivering therapeutic siRNA to target oncogenic driver genes [23]. The therapeutic NP is loaded with siRNA that is capable of downregulating driver genes like mutant Kras and gastrin to inhibit growth of pancreatic cancer in orthotopic murine models [23,24]. The CCKBR serves as an anchor for the NP to bind and internalize releasing the payload intracellularly.

Radiolabeled imaging serves as a cornerstone for visualizing malignancies by exploiting specific tumor-targeting vectors to non-invasively track and quantify disease progression at the molecular level [33]. CCKBR scintigraphy with high detection rates in metastatic medullary thyroid cancer [34] demonstrates the profound clinical utility of this receptor in detection and staging. The stark, binary-like activation observed across the tumor cohort, even within early-Stage I specimens, indicates that *CCKBR* upregulation is a defining early-stage molecular switch in malignant transformation. Diagnostically, this differential expression provides a molecular window for early detection. It establishes a biological rationale for developing specific, non-invasive molecular imaging agents, such as radiolabeled gastrin analogues for positron emission tomography (PET), which could significantly improve the detection of localized lesions over conventional, non-specific imaging modalities. Early radiolabeled mini-gastrin strategies for CCKBR imaging failed due to poor metabolic stability, rapid clearance, and high renal toxicity, which limited clinical translation [35,36,37]. In contrast, newer biodegradable, receptor-targeted nanoparticle technology described in this work overcomes these limitations by ensuring high stability and achieving superior tissue-specific localization within pancreatic intraepithelial neoplasia. This nanomedicine platform has the potential for high levels of diagnostic specificity that routine 18F-FDG PET scanning, which often misses low-metabolism precancerous clusters, cannot match.

The therapeutic refractoriness of PDAC underscores the necessity of moving away from monotherapies toward intelligent combination strategies that strike multiple parallel survival networks simultaneously. For example, recent work highlights that co-targeting cell-cycle progression and epigenetic transcription elements using CDK4/6 and BET inhibitors can synergistically halt tumor growth and reverse epithelial-to-mesenchymal transition (EMT) [38]. Mirroring the biological advantage of multi-pathway blocking, our group has targeted the CCKBR proliferation axis using the receptor antagonist proglumide in combination with standard chemotherapy regimens. This combination strategy has demonstrated powerful antitumor synergy in preclinical mouse models [28] and has been advanced in a Phase 1 human clinical trial [39]. Our dual-compartment approach successfully drives synergistic improvements in overall survival while actively downregulating EMT markers and suppressing distant metastatic progression in vivo.

Although a direct biochemical link mapping the precise upstream genomic kinetics by which the rs1800843 A allele triggers the initial alternative splicing choice has not been captured via minigene reporter assays in the current study, the downstream functional oncogenic mechanism of this splice variant is well-established. Our prior work explicitly demonstrated that overexpressing the exact 69-amino-acid sequence resulting from intron-4 retention within the third intracellular loop of the CCKBR directly drives accelerated cell proliferation and signaling activation [16]. Thus, while a distinct gap remains regarding the upstream molecular mechanics of allele-driven transcript processing, the downstream functional consequence of this structural receptor modification on pancreatic cancer biology is definitively supported by established experimental models. Future execution of in vitro minigene splicing reporter assays will serve as a valuable secondary direction to fully map the upstream regulatory kinetics of the rs1800843 locus.

The single-nucleotide polymorphism rs1800843, located within the *CCKBR* gene on chromosome 11 (position chr11:6270835, GRCh38.p14), serves as a critical genetic variant linked to pancreatic cancer vulnerability. High-risk screening panels governed by consortiums like the International Pancreatic Cancer Screening Consortium rely on BRCA1/2, PALB2, and CDKN2A because their high penetrance justifies aggressive, costly imaging surveillance like annual MRI or Endoscopic Ultrasound (EUS). Conversely, the CCKBR SNP is optimized as a stratification tool; its higher prevalence means it should not be used alone to trigger imaging at this time, but it can be paired with familial history to catch aggressive phenotypes earlier. Given its pronounced enrichment in pancreatic cancer patients relative to its general global carrier baseline, integrating the rs1800843 genotype into multi-gene screening panels alongside high-penetrance drivers like BRCA2 holds substantial promise for refining risk stratification and identifying individuals predisposed to highly aggressive oncogenic phenotypes.

While no direct microRNA perturbation or experimental knockdown assays were performed in the present study, the established external literature supports a highly plausible epigenetic framework that may explain our data. The mechanism of action controlling the expression of the CCKBR has been studied and involves epigenetic regulation by microRNAs (miR). Both miR148a and miR148b are dysregulated in obese individuals by DNA methylation [40,41,42,43], and obesity and type-2 diabetes have also been associated with an increased risk for PDAC [44,45]. A high-fat diet or obesity accelerates this process by inducing DNA methyltransferases (DNMT1/3b), resulting in hypermethylation and silencing the miR-148a promoter [46]. The increased expression of the *CCKBR* during carcinogenesis is inversely mediated by miR148 levels [47]. During pancreatic carcinogenesis in the mutant *KRAS* mouse models, levels of miR-148a decrease [48] as *CCKBR* expression increases [49], suggesting that miR148 serves as a tumor suppressor preventing the expression of CCKBR. At the same time that *CCKBR* expression increases during PanIN progression, miRNA-148 decreases [48]. The 3′-UTR of *CCKBR* has two sites in conserved and poorly conserved regions that can bind with the seed region of members of the miR-148 family [50]. This combined mechanism creates a synergistic, highly oncogenic environment, where metabolic stress directly drives the molecular progression of PDAC.

CCKBRs have also been identified on pancreatic stellate cells (PSCs) [51]—the cells responsible for the dense fibrosis in the PDAC microenvironment. Pharmacologic blockade of CCKBR on PSCs with the oral CCKBR antagonist proglumide has been shown to dismantle the fibroblastic microenvironment in murine models [28] and by inducing pancreatic stellate cell (PSC) plasticity [31] and reducing extracellular matrix components (collagen 1α1 and αSMA). The reduction in tumoral fibrosis by proglumide facilitated the influx of CD8+ cytotoxic T cells while reducing immunosuppressive M2 macrophages [28]. These preclinical findings were successfully translated into a Phase 1 clinical trial of metastatic PDAC, where patient biopsies confirmed that CCKBR blockade with proglumide combined with chemotherapy effectively remodels the tumor microenvironment by decreasing cancer cell proliferation and fibrosis while promoting a favorable immune cell signature [39].

The distribution of the CCKBR is also present in the stomach and the brain [52,53], particularly in the cerebral cortex, where it acts as a primary neurotransmitter receptor regulating anxiety and nociception. In preclinical studies, no evidence of stomach uptake of the CCKBR-targeted nanoparticles or inflammation was found in the stomach histologically [23]. Ex vivo organ imaging and immunohistochemical evaluation confirmed that fluorescence from the nanoparticles localized uniquely to the altered pancreas. No evidence of nanoparticle uptake or signal was detected in alternative organs. Studies have confirmed that polyplex micelles are structurally blocked by the blood–brain barrier and therefore will not interact with brain tissue [54]. Consequently, these findings highlight the excellent systemic safety profile and high target specificity of the polyplex micelle delivery system.

## 5. Conclusions

In conclusion, these data establish CCKBR as a high-potential, dual-purpose biomarker for early precision diagnostics and targeted stromal-disrupting therapeutics in pancreatic cancer. The transcriptional divergence of CCKBR between healthy tissue and primary tumors underscores its potential utility as both a diagnostic biomarker and a selective therapeutic vulnerability in pancreatic adenocarcinoma. The lack of uptake of CCKBR-targeted NPs in other tissues supports the potential for decreased toxicity in other tissues. From a therapeutic perspective, the expression gap between malignant cells and normal pancreatic architecture minimizes the risk of off-target toxicities, presenting an opportunity for targeted interventions. Because CCKBR activation triggers downstream proliferative pathways and sustains an aggressive, desmoplastic tumor microenvironment through pancreatic stellate cell cross-talk, disrupting this axis holds significant therapeutic promise. This targetable profile can be exploited through small-molecule receptor antagonists (such as proglumide), antibody-drug conjugates, or peptide receptor-targeted nanomedicine designed to selectively deliver cytotoxic payloads directly to the tumor mass while sparing healthy tissues. Consequently, these findings reposition CCKBR from a poorly understood gastrointestinal receptor to a high-priority, actionable candidate for precision oncology frameworks in pancreatic cancer.

## 6. Patents

This work is supported by two US patents: US 11246881-B2, “Nanoparticle to target cancer” and US 2024/0252696-A1, “Targeting the cholecystokinin-B receptor for imaging and early detection of pancreatic cancer and pre-cancerous lesions.”

## Figures and Tables

**Table 1:** Key resource table.

Reagent or Resource	Source/Repository	Identifier/Reference
Gene Target Name	Ensembl	*CCKBR*
Ensembl Gene ID	Ensembl Release 104	ENSG00000110148.9
Primary Isoform ID	GENCODE/Ensembl	ENST00000334619.6 (CCKBR-001)
Tumor Cohort Source	The Cancer Genome Atlas (TCGA)	TCGA-PAAD (*n* = 178)
Healthy Control Cohort	Genotype-Tissue Expression (GTEx)	GTEx Pancreas (*n* = 328)
Analysis Platform	Peking University (Zhang Lab)	GEPIA2 Portal (v2.0)
Statistical Engine	SciPy/Statsmodels	scipy.stats (v1.12+)

## Data Availability

All data are available on public websites, on-line resources and in this publication, and the raw data for calculating the normal pancreas (GTEx) versus pancreatic cancer (TCGA) CCKBR expression are provided in the Supplemental Data.

## Supplementary Materials

The following supporting information can be downloaded at: https://www.mdpi.com/article/10.3390/cancers18213551/s1, Table S1: CCKBR gene expression levels from GTEx (normal pancreas) and TCGA (pancreatic ductal adenocarcinoma); Table S2: Cox proportional hazards model of the unified individual patient data (IPD) matrix (*N* = 1080).

## Abbreviations

The following abbreviations are used in this manuscript:

CCKBR	Cholecystokinin-B receptor
KC	p48-Cre/LSL-Kras^G12D^ transgenic Kras mouse
TCGA	The Cancer Genome Atlas
GTEx	Genotype-Tissue Expression repository
SNP	Single-nucleotide polymorphism
PDAC	Pancreatic ductal adenocarcinoma
PSCs	Pancreatic stellate cells
PanIN	Pancreatic intraepithelial neoplasia
ROC	Receiver operating characteristic
IVIS	In vivo optical imaging system
NAT	Normal adjacent tumor
