# Supplementary material for: The Cholecystokinin-B Receptor as a Candidate Diagnostic Biomarker and Translational Target for Precision Oncology in Pancreatic Cancer
Source: Cancers (Basel). 2026 Nov 5;18(21):3551. doi: 10.3390/cancers18213551 (PMC13762120; doi:10.3390/cancers18213551)
Supplement: Supplementary file 1 [file cancers-18-03551-s001.zip › cancers-4638987-supplementary.pdf]

Table S1: CCKBR gene expression levels from GTEx (normal pancreas) and TCGA (PDAC)

| Normal Pancreas | Pancreatic cancer |
|---|---|
| 0.41 | 5.4 |
| 0.55 | 3.12 |
| 0.32 | 8.65 |
| 0.78 | 14.21 |
| 0.64 | 1.95 |
| 0.51 | 4.77 |
| 0.89 | 12.1 |
| 0.44 | 6.34 |
| 0.37 | 2.88 |
| 0.62 | 9.5 |
| 0.71 | 18.45 |
| 0.5 | 4.1 |
| 0.46 | 7.23 |
| 0.82 | 3.89 |
| 0.6 | 11.56 |
| 0.39 | 6.9 |
| 0.58 | 1.45 |
| 0.94 | 5.12 |
| 0.67 | 16.32 |
| 0.43 | 2.34 |
| 0.52 | 10.78 |
| 0.35 | 4.65 |
| 0.73 | 7.89 |
| 0.81 | 13.4 |
| 0.49 | 5.01 |
| 0.61 | 3.67 |
| 0.56 | 9.12 |
| 0.4 | 22.45 |
| 0.76 | 6.54 |
| 0.88 | 1.12 |
| 0.31 | 5.89 |
| 0.65 | 11.23 |
| 0.47 | 4.34 |
| 0.53 | 8.76 |
| 0.7 | 15.9 |
| 0.42 | 3.45 |
| 0.59 | 7.1 |
| 0.36 | 12.67 |
| 0.84 | 5.23 |
| 0.69 | 9.88 |
| 0.48 | 2.11 |
| 0.72 | 6.78 |
| 0.54 | 14.56 |
| 0.38 | 4.9 |
| 0.63 | 3.25 |

| | |
|---|---|
| 0.91 | 8.43 |
| 0.45 | 19.12 |
| 0.57 | 7.34 |
| 0.75 | 5.67 |
| 0.83 | 11.01 |
| 0.28 | 3.78 |
| 0.66 | 6.21 |
| 0.5 | 2.95 |
| 0.41 | 10.45 |
| 0.77 | 7.56 |
| 0.61 | 1.89 |
| 0.34 | 5.6 |
| 0.85 | 13.89 |
| 0.52 | 8.45 |
| 0.49 | 4.12 |
| 0.74 | 12.34 |
| 0.39 | 5.1 |
| 0.68 | 8.23 |
| 0.92 | 16.12 |
| 0.46 | 6.01 |
| 0.53 | 3.9 |
| 0.79 | 9.78 |
| 0.62 | 25.3 |
| 0.33 | 7.12 |
| 0.87 | 1.56 |
| 0.58 | 6.45 |
| 0.7 | 11.9 |
| 0.4 | 4.78 |
| 0.64 | 9.32 |
| 0.51 | 17.45 |
| 0.47 | 3.78 |
| 0.8 | 7.67 |
| 0.35 | 14.12 |
| 0.73 | 5.89 |
| 0.69 | 10.34 |
| 0.44 | 2.45 |
| 0.56 | 7.34 |
| 0.86 | 15.23 |
| 0.31 | 5.34 |
| 0.67 | 3.6 |
| 0.55 | 8.99 |
| 0.42 | 20.56 |
| 0.71 | 7.89 |
| 0.9 | 6.21 |
| 0.59 | 11.89 |
| 0.37 | 4.23 |
| 0.63 | 6.76 |

| | |
|---|---|
| 0.48 | 3.34 |
| 0.78 | 11.21 |
| 0.52 | 7.99 |
| 0.45 | 2.12 |
| 0.82 | 6.12 |
| 0.6 | 15.1 |
| 0.36 | 8.9 |
| 0.75 | 4.67 |
| 0.5 | 13.12 |
| 0.89 | 5.56 |
| 0.43 | 17.56 |
| 0.66 | 6.45 |
| 0.54 | 4.23 |
| 0.38 | 10.45 |
| 0.72 | 21.9 |
| 0.61 | 7.56 |
| 0.49 | 1.98 |
| 0.84 | 6.99 |
| 0.29 | 12.54 |
| 0.57 | 5.21 |
| 0.76 | 9.87 |
| 0.41 | 18.9 |
| 0.68 | 4.12 |
| 0.53 | 8.12 |
| 0.81 | 15.43 |
| 0.46 | 6.34 |
| 0.65 | 10.95 |
| 0.93 | 2.78 |
| 0.34 | 7.89 |
| 0.7 | 16.1 |
| 0.58 | 5.78 |
| 0.51 | 3.95 |
| 0.39 | 9.54 |
| 0.74 | 23.12 |
| 0.62 | 8.45 |
| 0.47 | 6.76 |
| 0.85 | 12.54 |
| 0.67 | 4.67 |
| 0.4 | 7.32 |
| 0.63 | 3.78 |
| 0.79 | 11.98 |
| 0.32 | 8.43 |
| 0.56 | 2.45 |
| 0.88 | 6.65 |
| 0.44 | 16.45 |
| 0.69 | 9.45 |
| 0.52 | 5.23 |

| | |
|---|---|
| 0.36 | 14.1 |
| 0.73 | 6.12 |
| 0.48 | 9.34 |
| 0.6 | 19.1 |
| 0.83 | 6.9 |
| 0.45 | 4.67 |
| 0.71 | 11.23 |
| 0.55 | 24.56 |
| 0.38 | 8.12 |
| 0.8 | 2.34 |
| 0.62 | 7.56 |
| 0.59 | 13.21 |
| 0.42 | 5.67 |
| 0.76 | 10.45 |
| 0.68 | 20.34 |
| 0.35 | 4.56 |
| 0.9 | 8.67 |
| 0.51 | 16.9 |
| 0.47 | 6.89 |
| 0.64 | 11.56 |
| 0.82 | 3.12 |
| 0.3 | 8.45 |
| 0.75 | 17.23 |
| 0.53 | 6.23 |
| 0.41 | 4.34 |
| 0.69 | 10.12 |
| 0.46 | 21.89 |
| 0.86 | 9.12 |
| 0.61 | 7.34 |
| 0.39 | 13.21 |
| 0.72 | 5.12 |
| 0.54 | 7.89 |
| 0.81 | 4.23 |
| 0.43 | 12.67 |
| 0.65 | 8.9 |
| 0.5 | 2.78 |
| 0.87 | 7.12 |
| 0.34 | 17.89 |
| 0.78 | 9.98 |
| 0.58 | |
| 0.49 | |
| 0.66 | |
| 0.37 | |
| 0.7 | |
| 0.52 | |
| 0.44 | |
| 0.83 | |

| | |
|---|---|
| 0.61 | |
| 0.4 | |
| 0.74 | |
| 0.56 | |
| 0.91 | |
| 0.47 | |
| 0.63 | |
| 0.55 | |
| 0.33 | |
| 0.77 | |
| 0.68 | |
| 0.42 | |
| 0.85 | |
| 0.27 | |
| 0.73 | |
| 0.51 | |
| 0.46 | |
| 0.8 | |
| 0.64 | |
| 0.38 | |
| 0.71 | |
| 0.53 | |
| 0.89 | |
| 0.45 | |
| 0.62 | |
| 0.57 | |
| 0.35 | |
| 0.76 | |
| 0.69 | |
| 0.48 | |
| 0.82 | |
| 0.6 | |
| 0.41 | |
| 0.74 | |
| 0.52 | |
| 0.84 | |
| 0.39 | |
| 0.66 | |
| 0.58 | |
| 0.47 | |
| 0.7 | |
| 0.92 | |
| 0.33 | |
| 0.55 | |
| 0.79 | |
| 0.43 | |
| 0.61 | |

| | |
|---|---|
| 0.5 | |
| 0.86 | |
| 0.46 | |
| 0.73 | |
| 0.65 | |
| 0.36 | |
| 0.68 | |
| 0.54 | |
| 0.4 | |
| 0.77 | |
| 0.59 | |
| 0.49 | |
| 0.81 | |
| 0.63 | |
| 0.34 | |
| 0.75 | |
| 0.52 | |
| 0.88 | |
| 0.45 | |
| 0.67 | |
| 0.56 | |
| 0.42 | |
| 0.72 | |
| 0.9 | |
| 0.58 | |
| 0.38 | |
| 0.64 | |
| 0.48 | |
| 0.83 | |
| 0.51 | |
| 0.44 | |
| 0.79 | |
| 0.6 | |
| 0.37 | |
| 0.76 | |
| 0.53 | |
| 0.85 | |
| 0.43 | |
| 0.69 | |
| 0.55 | |
| 0.39 | |
| 0.71 | |
| 0.62 | |
| 0.5 | |
| 0.8 | |
| 0.31 | |
| 0.57 | |

| | |
|---|---|
| 0.74 | |
| 0.41 | |
| 0.66 | |
| 0.53 | |
| 0.87 | |
| 0.46 | |
| 0.68 | |
| 0.91 | |
| 0.35 | |
| 0.7 | |
| 0.59 | |
| 0.48 | |
| 0.78 | |
| 0.61 | |
| 0.43 | |
| 0.84 | |
| 0.65 | |
| 0.39 | |
| 0.73 | |
| 0.54 | |
| 0.81 | |
| 0.32 | |
| 0.56 | |
| 0.89 | |
| 0.45 | |
| 0.67 | |
| 0.52 | |
| 0.4 | |
| 0.76 | |
| 0.5 | |
| 0.63 | |
| 0.82 | |
| 0.47 | |
| 0.7 | |
| 0.58 | |
| 0.36 | |
| 0.85 | |
| 0.62 | |
| 0.55 | |
| 0.42 | |
| 0.74 | |
| 0.69 | |
| 0.34 | |
| 0.88 | |
| 0.53 | |
| 0.49 | |
| 0.46 | |

| | |
|---|---|
| 0.71 | |

**Table S2. Cox Proportional Hazards Model of the Unified Individual Patient Data (IPD) Matrix (N=1,080)**

| Study Source / Baseline Stratification Factor | Sample Size (N) | Hazard Ratio (HR) | 95% Confidence Interval |
|---|---|---|---|
| Cohort 1: PMID: 22277584 *(Cleaned)* | 27 | 3.12 | [1.65, 5.90] |
| Cohort 2: PMID: 25469546 | 931 | 1.83 | [1.52, 2.21] |
| Cohort 3: PMID: 26646278 | 122 | 1.04 | [0.72, 1.51] |
| Unified IPD Pooled Model (Figure 2d) | 1,080 | 1.78 | [1.51, 2.10] |

Note: Baseline study-level heterogeneity metrics assessed prior to matrix unification: $Q = 10.85$, $df = 2$, $p = 0.0044$; $I^2 = 81.6\%$. The single, primary individual patient data (IPD) analysis combines all cohorts after the deduplication of 24 overlapping records, yielding the final aggregate hazard ratio of 1.78 (Log-Rank $p = 1.42 \times 10^{-11}$) presented visually in Figure 2d.
